# The Rehabilitation and the Robotics: Are They Going Together Well?

**DOI:** 10.3390/healthcare9010026

**Published:** 2020-12-30

**Authors:** Daniele Giansanti

**Affiliations:** Centre Tisp, Istituto Superiore di Sanità, Via Regina Elena 299, 00161 Roma, Italy; daniele.giansanti@iss.it; Tel.:+39-06-49902701

## 1. Rehabilitation and the Robotics

The following problems have always existed in rehabilitation [1]:

- Operational and functional reorganization from a cerebral point of view and motor recovery seem to require therapies that require an important use of the limb associated with an innovative type of learning and/or ability with regard to new motor skills.
- Based on the previous consideration, it is evident that simple movements do not lead to maximum recovery of the rehabilitated limb.
- Based on the first consideration, it is also clear that even the use of passive exercises does not lead to optimal recovery of the affected limb.

Hence the reasoning that led to the genesis and first use of robotics as practical and effective rehabilitation tools [2,3] because they can allow administration of rehabilitation therapies that include:

1. Motivating and engaging rehabilitation exercises.
2. Training that both optimizes and maximizes the functionality of the limb.
3. An environment full of motivating stimuli.

Rehabilitation supported by the use of robotic systems can have numerous advantages [4]. In particular, it allows more intensive and tailored to the patient rehabilitation activities and services (increasing the amount and quality of therapy that can be administered) and allows all the involved actors in the team (e.g., physiotherapists, physicians, bioengineers and other figures) to set and manage some work parameters to make the rehabilitation specific and optimal for the patient (the type of exercise, the level of assistance from the robot, the force and the kinematic that the patient must exert, following the exercize).

### 1.1. Robotic Technological Tools Used in Rehabilitation

There are two different types of robotic technological tool (RTT) in rehabilitation for both the lower and upper limbs. The first is based on exoskeletal instruments. The second is of the end-effector type.

#### 1.1.1. Exoskeletal-Type RTT

The exoskeletal robot, whether it is for the lower [5] or upper limbs [6], completely covers the limb, following and replicating its anthropometric characteristics and thus guiding each segment involved in the rehabilitation practice. The exoskeletons are systems with a mixture of mechanical and electronic components that constitute a mechatronic apparatus that is worn and that performs the same type of kinematic/dynamic activity practiced by the patient who wears it. These systems cover the affected limb, or at least the part of the limb affected by the clinical aspects from a rehabilitation point of view. In these systems the number of degrees of freedom is equal to that of the joints on which the rehabilitation therapy must intervene based on the objectives. Regarding the rehabilitation of the lower limbs [4] we refer to class 1 exoskeletal systems in reference to nonportable robotic systems. Class 1 belong to those nonportable robotic systems consisting of a robotic exoskeleton. In some cases there is also a body weight support (BWS) [4] type system distributed over the whole body for weight relief, a conveyor belt and a control information system including biofeedback response systems based on virtual reality. These systems are naturally used only in the clinic and partly constitute an evolution of pure BWS systems. We expressly refer to Class 2 exoskeletal systems with specific reference to portable systems that can also be used externally to the rehabilitation clinical environment.

#### 1.1.2. End-Effector-Type RTT

In a robotic end-effector device, the input for carrying out the rehabilitation exercise comes directly from the distal part of the limb, allowing the natural kinematic activation of the movement without unnatural constraints. These systems are used for both lower [7] and upper limb [8,9] rehabilitation. The robot with the end-effector interconnects to the limb in a single point, generally a handle or a grip point for the rehabilitation of the upper limb or a pedal-like tool for the rehabilitation of the lower limbs. As regards the rehabilitation of the upper limbs with reference to end-effector systems, in some cases we speak of Cartesian systems due to some constraints that can be imposed in the trajectories also combined with specific exercises (also gamified) provided by software.

### 1.2. Beneficts of the RTTs

Both the two RTTs produce patient benefits [4,5,6,7,8,9].

It is now well established that for the lower limbs the RTT produces various benefits, including:

- Improved trunk control.
- Improvement of the sleep-wake rhythm and reduction of perceived fatigue in carrying out daily life activities.
- Pain relief.
- Improvement in the state of mental health.
- Improvement of general anthropometric characteristics (reduction of fat mass, increase of lean mass).
- Improvement of intestinal and bladder function.

Some of these benefits are also obtained thanks to combination with specific software also based on virtual reality (VR) and/or augmented reality (AR), and also in defined protected immersive virtual environments where the rehabilitation scenarios called Cave Automatic Virtual Environment (better known with the acronym CAVE) take place.

It is now well established that for the upper limbs the use of an RTT shows several benefits, including:

- Neuromotor improvement of limb function.
- Pain relief.
- Improvement in the state of mental health
- Improvement of general anthropometric characteristics (reduction of fat mass, increase of lean mass)
- Improvement of cognitive functions.

Some of these benefits are also obtained thanks to the combination with specific software that generally offer motivating GAME and recently, in some cases, also based on virtual reality (VR) and/or augmented reality (AR).

## 2. New Directions to Explore and Open Problems: Aims of the Editorial

### 2.1. New Directions of Research and Development and First Aim of the Editorial

Currently, robotics for rehabilitation are pushing a lot of research and development and numerous new interesting directions are opening both directly connected to the robotic tools mentioned above and in support of an even wider rehabilitation process.

Some of these directions that more directly relate with motion rehabilitation [4,10,11] are:

1. To assess the effects of using robots at different phases of recovery.
2. To develop wearable robots easy and practical to wear and remove.
3. To decrease the costs also by means of new models of care.
4. To optimize and rethink the models of care based on robotics.
5. To empower the synergy and collaboration between professionals of the rehabilitation team and designers through shared and properly designed projects.
6. To make virtual reality, augmented reality, at home technologies, exoskeleton and artificial intelligence available for the treatment of cognitive and/or degenerative conditions.

Other directions, more focused to psychological support, in a wider approach to rehabilitation process are the following [12]:

1. To invest in social robots specifically designed to support during the rehabilitation phases (as for example in the care of the elderly).
2. To invest in social robots specifically designed as cultural mediators to support during communication/therapy activity (as in the care of the autism).
3. To face the problem of the empathy in robotics especially in relation to interaction with the social robots.

In light of the above, the editorial aims to stimulate scholars to report their experiences relating to various aspects of innovation on the development and use of robotics in rehabilitation both from a technological and clinical point of view. For all the above listed issues, and in particular for the 6, 7 and 8, perspective articles are welcome.

### 2.2. Open Problems and Second Aim of the Editorial

Despite the great development of robotics in the rehabilitation field, we are assisting to several different approaches in the use and in the relevant models of care. For example, both the rehabilitation therapies and the outcomes in the international panorama are often assessed in a different way. As in other sectors, such as telemedicine, robotics is often used very limited to pilot and/or research projects. Just like in telemedicine, all aspects that can strengthen the use of robotics in routine clinical activities must be addressed in the international panorama with strong dedicated initiatives. Through this approach, rehabilitation robotics will be able to be part of the portfolio of proposed healthcare offers in every state with a clear reimbursement of the indicated services. In light of the above, the editorial aims also to stimulate scholars to report their experiences related to these various aspects of the use of robotic technologies used in the rehabilitation centers and laboratories. From this collection obtained with heterogeneous methods, that presumably will range from the review to the mass survey, we expect to have important responses and stimuli for the international scientific community.
